# Synthesis and Study of Thermoresponsive Amphiphilic Copolymers via RAFT Polymerization

**DOI:** 10.3390/polym14020229

**Published:** 2022-01-06

**Authors:** Marija Kavaliauskaite, Medeina Steponaviciute, Justina Kievisaite, Arturas Katelnikovas, Vaidas Klimkevicius

**Affiliations:** Institute of Chemistry, Vilnius University, LT-03225 Vilnius, Lithuania; ma.kavaliauskaite@gmail.com (M.K.); medeina.steponaviciute@chf.vu.lt (M.S.); justinakevisaite@gmail.com (J.K.); arturas.katelnikovas@chf.vu.lt (A.K.)

**Keywords:** RAFT polymerization, kinetics, amphiphilic, azeotropic, stimuli-response copolymers, conformational changes

## Abstract

Synthesis and study of well-defined thermoresponsive amphiphilic copolymers with various compositions were reported. Kinetics of the reversible addition-fragmentation chain transfer (RAFT) (co)polymerization of styrene (St) and oligo(ethylene glycol) methyl ether methacrylate (PEO_5_MEMA) was studied by size exclusion chromatography (SEC) and ^1^H NMR spectroscopy, which allows calculating not only (co)polymerization parameters but also gives valuable information on RAFT (co)polymerization kinetics, process control, and chain propagation. Molecular weight *M_n_* and dispersity *Đ* of the copolymers were determined by SEC with triple detection. The detailed investigation of styrene and PEO_5_MEMA (co)polymerization showed that both monomers prefer cross-polymerization due to their low reactivity ratios (r_1_ < 1, r_2_ < 1); therefore, the distribution of monomeric units across the copolymer chain of *p*(St-*co*-PEO_5_MEMA) with various compositions is almost ideally statistical or azeotropic. The thermoresponsive properties of *p*(St-*co*-PEO_5_MEMA) copolymers in aqueous solutions as a function of different hydrophilic/hydrophobic substituent ratios were evaluated by measuring the changes in hydrodynamic parameters under applied temperature using the dynamic light scattering method (DLS).

## 1. Introduction

Over the past few decades, multifunctional polymers have received significant interest and recognition. Among them, ever-increasing attention has been focused on stimuli-responsive (smart/intelligent/environmentally sensitive) polymers [1]. The most important feature of such polymers is dramatic and abrupt conformational and/or chemical alterations upon exposure to internal and/or external chemical/physical stimuli. Applied stimuli could affect the polymer solubility, formation of an intricate molecular self-assembly, or a sol-to-gel transition. Different functional groups in the polymer respond to different stimuli, and the most utilized ones are pH, temperature, mechanical stress, the presence of various molecules and biomolecules, electric/magnetic fields [2,3]. For instance, thermoresponsive polymers in solution adopt an expanded coil conformation, where at the phase separation temperature they collapse to form compact globuli. pH-responsive polymers, in turn, undergo conformational changes in response to solution pH variation, as a result of protonation or deprotonation of functional groups in polymer chains. Furthermore, ester or amide functional groups in the enzyme-responsive polymers can be cleaved in presence of specific enzymes resulting in changes of polymer solubility, morphology, and solvation state [3].

They can also have various architectures (e.g., diblock, stars, brushes) and be encountered as independent macromolecules, supramolecular assemblies, coatings, or a combination of several mentioned structures. Moreover, it is also crucial that stimuli-response is reversible, i.e., polymer returns to its initial phase after the counter trigger application [4]. Due to such unique properties, the stimuli-responsive polymers can be applied in various fields, for instance, sensing and bio-sensing [5], drug delivery [6,7], artificial muscles [8,9], etc.

Stimuli-responsive behavior is essentially dictated by the functional groups present within the polymer backbone or at the side chains. The responsivity itself is determined by the nature of functional groups, their distribution, and the composition of copolymer. It should also be mentioned that the responsivity is very sensitive to the polymer composition; therefore, it is crucial to synthesize polymers with desired and precise parameters [10]. Reversible-deactivation radical polymerization (RDRP) and its derivative methods (atom-transfer radical polymerization (ATRP) [11], reversible addition-fragmentation chain transfer (RAFT) polymerization [10], and nitroxide-mediated polymerization (NMP) [12]) are particularly suitable for the synthesis of such polymers since they offer a high degree of polymerization control. With the aid of these methods, stimuli-responsive polymers with required parameters, morphologies, and structure (for instance, linear polymers (homopolymers, multiblock copolymers, and organic/inorganic hybrid polymers) [13], and nonlinear polymers (star polymers, (hyper)branched polymers) [14]) can be designed and prepared. However, the tacticity of most copolymers formed by any RDRP techniques is difficult to predict. Depending on the reactivity of monomers, the distribution of monomeric units within copolymer chains could be statistical, gradient, alternating, pseudo-block, etc. When stimuli-responsive polymers are designed, it is particularly important to have the functional groups/substituents statistically distributed within copolymer chains. In such cases, the reproducibility of stimuli-responsive properties of copolymers could be ensured independently of polymer conversion. This, however, can only be achieved if the monomer reactivity ratios are very close (e.g., copolymerization of two similar monomers) [15].

Although such stimuli-responsive polymers can be synthesized by all mentioned RDRP methods, the RAFT method has several advantages over the others. The RAFT method offers low dispersity, different functionality, and relatively simple polymerization conditions, ensuring the repeatability, reliability, and uniform response of the target polymers [10]. There are many different polymers reacting to different stimuli; however, the most studied and best understood among them is the temperature response (or “thermo-response”) polymers. Their phase transition from clear to unclear in aqueous solutions is observed if the temperature of polymer solution is higher than the lower critical solution temperature (LCST). To this day, the largest number of scientific papers on the synthesis and application of intelligent polymers are intended for thermo-responsive materials [16,17].

The most extensively studied polymer with LCST is homopolymer poly(*N*-isopropylacrylamide) (pNIPAM) or other copolymers with this monomer [18,19,20,21,22]. It is known that LCST of pNIPAM is 32 °C and it is close to the human physiological temperature [18]. When the solution temperature rises above the LCST, the pNIPAM chains change from solvated (bound to water molecules) or extended random coil to globular conformation [19]. These changes of polymer architecture can be easily controlled by changing polymer composition, i.e., by copolymerization with different hydrophilic or hydrophobic monomers, i.e., the LCST temperature can be increased or decreased. However, it is often forgotten that the main reason for such popularity of thermo-responsive polymers is not the LCST value itself (since other polymers exhibit LCST values even closer to 37 °C) but the fact that the LCST is relatively insensitive to other environmental factors, such as pH, concentration, or chemical stimulus [19,20]. The changes of these environmental factors impact the LCST of pNIPAM only by few degrees. Another important factor that has made pNIPAM so popular is the belief that it is bio-inert to other materials. However, Schild et al. [21] showed that this is not entirely true: due to a large number of secondary amide groups in the polymer chain, such polymer can irreversibly interact with proteins via hydrogen bonds [22]. Moreover, the phase transformations of this polymer are not infinite and, after a certain number of cycles, they become irreversible [21].

In addition to pNIPAM, some other polymers with LCSTs properties were also recently reported: poly(*N*,*N*-dimethylaminoethyl methacrylate) (DMAEMA) [23], poly(2-(*N*-morpholine)ethyl methacrylate) (MEMA) [24], poly(*N*,*N*-diethylaminoethyl methacrylate) (DEAEMA) [25], poly(*N*-[2-(diethylamino)ethyl acrylamide) (DEAEAM) [26], poly(*N*,*N*-diethylacrylamide) (DEAAM) [27], and poly(ethylene glycol) methyl ether methacrylate (PEOMEMA) [28]. Among this list, the PEOMEMA compound is the one that has the greatest potential to become superior to NIPAM, which can be considered as the “gold standard” of thermoresponsive polymers so far. Copolymerization with oligo- (ethylene glycol) macromonomers containing different chain-lengths (i.e., of different hydrophilicity but similar chemical nature) can help creating thermosensitive copolymers with a tunable LCST. Moreover, oligo-(ethylene glycol) macromonomers are neutral, water-soluble, non-toxic, and the most applied synthetic polymer in the biomedical field [29,30]. For example, the LCST of 32, 37, or 39 °C was observed in pure water for *p*(MEO_2_MA-*co*-OEGMA) copolymers possessing on average 5, 8, or 10 mol% OEGMA units, respectively [29].

The main task of the present work was to analyze RAFT copolymerization of hydrophobic styrene and thermoresponsive poly(ethylene glycol) methyl ether methacrylate (PEO_5_MEMA). In this paper we also clarify the monomers attachment to the polymer chain mechanism, which would allow the synthesis of thermosensitive polymers of the desired composition and architecture. Another part of the work was designated to investigate these copolymers and evaluate the resulting polymers’ thermal sensitivity properties.

## 2. Materials and Methods

### 2.1. Materials

Poly(ethylene oxide) monomethyl ether methacrylate (PEO_5_MEMA, *M_n_* 300, Aldrich, Saint Louis, MO, USA) was purified from inhibitors by passing through a chromatographic column filled with basic alumina (Type 5016A, Fluka, Seelze, Germany). Styrene (*M_n_* 104.15, Aldrich, Saint Louis, MO, USA) was distilled under reduced pressure before use. 1,4-dioxane (DO, 99.8%) and other solvents (ethyl acetate, hexane, toluene, tetrahydrofuran (THF), diethyl ether) were purchased from Eurochemicals and used without further purification unless specified otherwise. 4,4-Azobis(4-cyanovaleric acid) (ACVA, 98%, Fluka, Seelze, Germany), carbon disulfide (CS_2_, 99.9%, Aldrich, Saint Louis, MO, USA), 1-butanthiol (99%, Aldrich, Saint Louis, MO, USA), sodium hydride (NaH, 60% dispersion in mineral oil, Aldrich, Saint Louis, MO, USA), iodine (I_2_, 99.8%, Aldrich, Saint Louis, MO, USA) were used as received. 2,2′-Azobisisobutyronitrile (AIBN) (Chempur, Piekary Slaskie, Poland) was purified by recrystallization from methanol (twice). RAFT chain transfer agent 4-(((butylthio)carbonothioyl)thio)-4-cyanopentanoic acid (CTA) was synthesized before copolymerization based on the previously published paper [31]. The detailed synthesis procedure and identification of used CTA is provided in ESI (S1).

### 2.2. RAFT Polymerization of Styrene

RAFT polymerization of styrene was carried out in 1,4-dioxane with the presence of 4-(((butylthio)carbonothioyl)thio)-4-cyanopentanoic acid and AIBN as chain transfer (CTA) agent and initiator, respectively. The typical procedure of styrene RAFT polymerization is presented below ([M]_0_:[CTA]_0_:[I]_0_ = 300:3:1, w([M]_0_) = 20%). Styrene (1.0 g, 9.6 mmol), CTA (28 mg, 0.096 mmol), and AIBN (5.25 mg, 0.032 mmol) were poured into a 25 mL round-bottomed flask with a magnetic stirrer and mixed with 3.2 g (3.1 mL) of 1,4-dioxane. Then the flask was flushed with nitrogen, capped, and placed in 80 °C thermostat for 48 h under vigorous stirring. After synthesis, the reaction was quenched by cooling the flask down to room temperature and opening it to air. The prepared polystyrene was purified by precipitation in methanol (twice). The precipitates were dried in a vacuum oven at 35 °C until constant weight. The product yield was determined gravimetrically (0.564 g, 56.4%).

### 2.3. RAFT Polymerization of p(St-co-PEO_5_MEMA)

RAFT copolymerization of styrene and PEO_5_MEMA at different molar ratios ([St]:[PEO_5_MEMA] = 100:0; 90:10, 80:20, 70:30; 60:40; 50:50, 40:60; 30:70, 20:80, 10:90, 0:100) was carried out in 1,4-dioxane. The initial molar ratio of the monomers to the CTA and the initiator was kept constant and equal to [M]_0_:[CTA]_0_:[I]_0_ = 300:3:1, and the synthesis procedure in all cases was the same. Polymerization was carried out in a round-bottomed flask under an inert atmosphere (N_2_) at 80 °C for 48 h. After synthesis, the reaction was quenched by cooling the flask down to room temperature and opening it to air. The copolymers, containing high amount of styrene units in composition, were purified by precipitation in methanol (twice) and dried under vacuum at 35 °C until constant weight. The copolymers with higher amount of PEO_5_MEMA, on the other hand, were dialyzed against DI water using 3.5 kDa MWCO tubes, concentrated via rotary evaporator and separated by freeze-drying. The product yield was determined gravimetrically.

### 2.4. Analysis and Characterization

^1^H and ^13^C NMR spectra were recorded on a Bruker 400 Ascend™ nuclear magnetic resonance spectrometer (400 MHz) in DMSO-d_6_ at 22 °C. FT-IR spectra were recorded with a Perkin Elmer spectrometer “Frontier Spectrum 100” with a 1 cm^−1^ resolution and making 15 scans. Raman spectra were obtained by Perkin Elmer spectrometer “Raman Station 400 F” using a near-infrared laser with the wavelength λ = 785 nm (max power exposed to sample—100 mW).

DLS measurements were performed on a Zetasizer Nano ZS (Malvern Instruments, Malvern, United Kingdom) with a 4 mW HeNe laser at a wavelength of 633 nm. The size distributions were obtained from the correlation functions and the data were analyzed using the Malvern Zetasizer software v. 7.03 (Malvern Instruments, Malvern, United Kingdom, accessed on the 14 September 2021).

The macromolecular parameters of the synthesized copolymers (number average and weight average molecular weights (*M_n_* and *M_w_*), and the dispersity (*Ð* = *M_w_*/*M_n_*)), were determined by size exclusion chromatography (SEC). SEC measurements were carried out in THF as an eluent at 30 °C using a flow rate of 0.5 mL/min., column Viscotek T6000 M General Mixed (Malvern Instruments, Malvern, United Kingdom), 300 × 8.0 mm. Viscotek TDAmax (Malvern Instruments, Malvern, United Kingdom) system for SEC measurements was equipped with a triple detection array (TDA305): a refractive index detector (RI); light scattering detector (LS), simultaneously measuring the scattered light (laser 3 mW, λ = 670 nm) at two angles—right-angle (90°) and low-angle (7°); and four-capillary bridge viscosity detector (DP). The system was calibrated using Viscotec PolyCAL^TM^ TDS-Mix-NB triple detection calibration standard (PS 99 K in THF). SEC data were processed using OmniSEC software v. 5.12 (Malvern Instruments, Malvern, United Kingdom, accessed on the 27 December 2021).

## 3. Results and Discussion

### 3.1. Study of Styrene Polymerization

**Figure 1:**
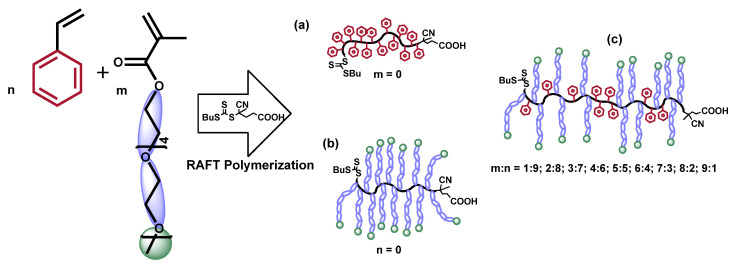
Principal synthesis scheme of *p*(St) (**a**), *p*(PEO_5_MEMA) homopolymers (**b**), and *p*(St-*co*-PEO_5_MEMA) copolymers with various compositions (**c**) via RAFT polymerization.

Polystyrene samples, used in this study, were prepared by RAFT polymerization method. RAFT polymerization method is versatile for the synthesis of polymers with different functionality and shows better polymerization control in comparison with other reversible deactivation radical polymerization (RDRP) methods (ATRP, NPM, etc.,) [32,33]. The second generation trithiocarbonate type CTA (4-(((butylthio)carbonothioyl)thio)-4-cyanopentanoic acid) was used for both styrene RAFT polymerization and controlled synthesis of amphiphilic copolymers. The principal polymerization and copolymerization of styrene and PEO_5_MEMA (*M_n_* = 300 g/mol) scheme is presented in Figure 1. As shown in our previous publications, the usage of such custom-made CTA in RAFT polymerization could ensure good polymerization control of more activated monomers (methacrylates) [31,34].

**Figure 2:**
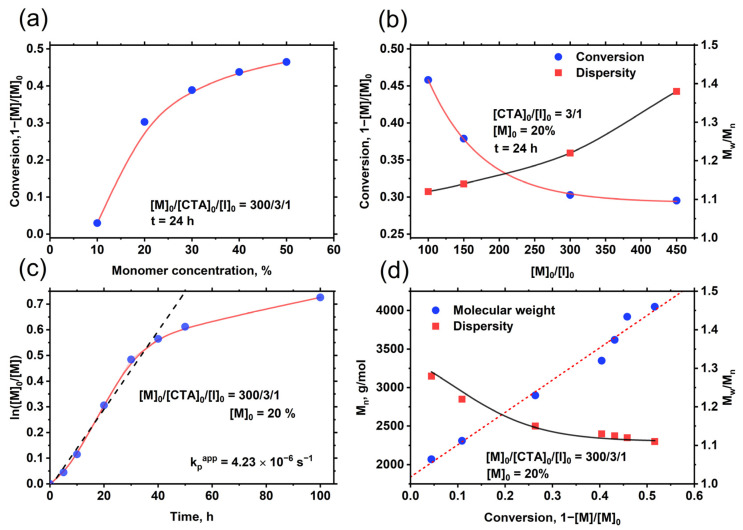
Dependence of monomer conversion during styrene RAFT polymerization using different monomer concentration (**a**) and initial molar ratio of monomer to initiator ([M]_0_/[I]_0_) (**b**). Pseudo first order kinetic plot (**c**); evolution of molecular weight and dispersity as a function of monomer conversion (**d**). Polymerization conditions: T = 70 °C, [CTA]_0_/[I]_0_ = 3/1, ([M]_0_ represents the monomer concentration in polymerization mixture).

The results of styrene polymerization in 1,4-dioxane using different synthesis parameters are presented in Figure 2. The provided data show that the conversion of styrene monomers is dependent on monomer concentration in the initial feed. Reducing monomer concentration from 50 to 10 wt.% ([M]_0_:[CTA]_0_:[I]_0_ = 300:3:1) resulted in a drastic drop of conversion from 46.5% to 3% (Figure 2a). However, the dispersity of polystyrene, in case of using diluted mixtures with monomer concentration of 20 wt.%, was as low as 1.17; whereas polymers obtained from more concentrated mixtures (50 wt.%) were more dispersed (*Ð* = 1.38) (molecular weight distribution curves of the synthesized polymers are presented in ESI (Figures S3–S5)). This behavior could be related to the mechanism of styrene RAFT polymerization (provided in ESI (Figure S2)), where the polymeric chains with random lengths formed during the initial step (conventional radical polymerization) could not be equalized as the main equilibrium of RAFT polymerization is achieved.

During the styrene polymerizations using different monomer to initiator ([M]_0_:[I]_0_) molar feed in the reaction mixture ([CTA]_0_:[I]_0_ ratio was maintained at 3), we have noticed that polymerizations of mixtures with higher [M]_0_:[I]_0_ ratios resulted in lower conversion and, most importantly, in lower polymerization control. For instance, when [M]_0_:[I]_0_ ratio in the initial reaction feed was changed from 100 to 450, the monomer conversion dropped from 45.8% to 29.5%, leading to an increase of obtained polymers dispersity from 1.12 to 1.38, respectively. Similar results are also presented in the literature, where several authors reported that the RAFT polymerization of styrene is complicated and results in low yields and poor control [35,36]. Ponnusamy et al. [36] reported polystyrene synthesis in bulk at 100 °C in the presence of different dodecyl-based CTAs. The conversion of monomers reached up to 48.8% and 52.6% during the polymerization process for 10 and 14 h, respectively. However, the dispersity of polymers reported by authors is relatively high (*Ð* = 1.38–1.48) [36]. Polystyrene synthesis via RAFT is unique if compared to the RAFT polymerization utilizing other “more active monomers” because the stability of the styrene radical is greater. Usually, secondary radicals are more reactive if compared to tertiary radicals (e.g., methacrylate); however, in the case of styrene, due to the π electrons in phenyl ring, the formed radical is delocalized through 7 carbon atoms, resulting in slower propagation rates. This effect can normally be counteracted by increasing the polymerization temperature and/or increasing the concentration of monomer.

Summarizing the results, it is evident that better RAFT polymerization control in the case of polystyrene synthesis is achieved using diluted reaction mixtures. However, in such particular cases, as expected, the conversion of monomers is low. Therefore, the remaining question is what is more important, better polymerization control or higher monomer conversion? Is it possible to increase the conversion of monomers by carrying out polymerizations in dilute solutions over a longer period? To answer these questions, we have performed several polystyrene syntheses in diluted reaction mixtures (20 wt.%) and quenched them after a different period of polymerization. The products were precipitated (twice) in methanol and dried to constant weight. Monomer conversion in all cases was determined gravimetrically. Macromolecular parameters of products, such as molecular weight and dispersity, were also evaluated using SEC. The obtained results are presented in Figure 2c,d. The molecular weight is linearly increasing with conversion, thus, dispersity shows the opposite behavior (as shown in Figure 2d). The dispersity of polystyrene samples at low monomer conversion is higher in comparison with those at higher monomer conversion. For instance, the dispersity measured for 4.4% (after 5 h) and 51.6% (after 100 h) of monomer conversion have values of 1.28 and 1.11, respectively. The ln([M_0_]/[M]) versus time of polymerization plots are shown in Figure 2c. The dependence is linear until 40 h of polymerization (43.1% of monomer conversion) and follows a pseudo-first-order kinetic equation. Styrene polymerization rate constant (k_p_^app^) is calculated from a linear trend (dashed line) in the kinetic plot (Figure 2c) and has a value of 4.23 × 10^−6^ s^−1^. The propagation step in styrene RAFT polymerization is relatively slow, the calculated polymerization constant is about 2 orders of magnitude lower if compared to other more-activated monomers (e.g., different methacrylates). Typically, the more activated monomers have similar values of polymerization constant; for example, PEGMA monomers, depending on the length of PEG side substituents, have k_p_^app^ values of 1.38–3.10 × 10^−4^ s^−1^ [34], whereas k_p_^app^ of HEMA is 1.75 × 10^−4^ s^−1^ [31].

### 3.2. Synthesis of p(St-co-PEO_5_MEMA) Copolymers

**Figure 3:**
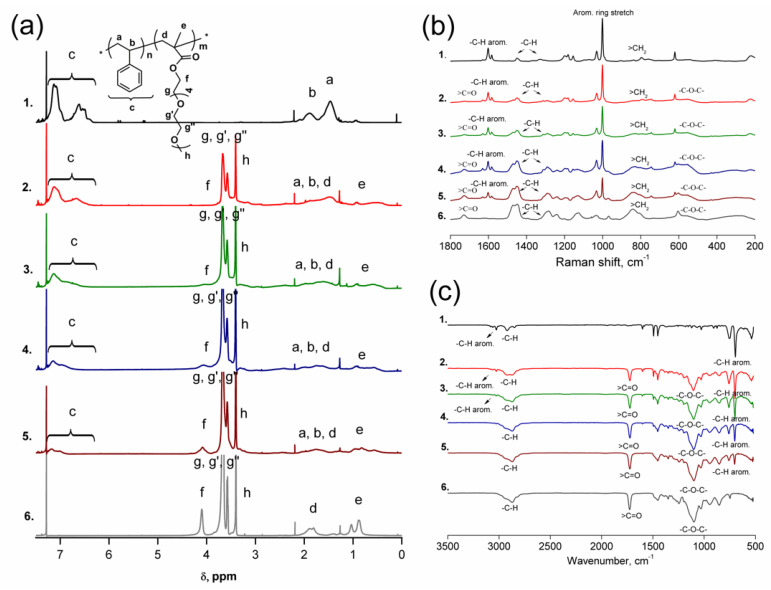
^1^H NMR (**a**), RAMAN (**b**), and FTIR (**c**) spectra of *p*(St) (1), *p*(PEO_5_MEMA) (6) and *p*(St-*co*-PEO_5_MEMA) copolymers with different composition. ([St]_0_:[PEO_5_MEMA]_0_: 8:2 (2) 6:4 (3), 4:6 (4), 2:6 (5).

After a detailed study of styrene RAFT homo-polymerization, the amphiphilic random *p*(St-*co*-PEO_5_MEMA) copolymers with different compositions of hydrophobic/hydrophilic segments were synthesized. The different composition of *p*(St-*co*-PEO_5_MEMA) copolymers was obtained by simple variation of styrene and PEO_5_MEMA molar feeds in the initial reaction mixture. The exact composition of synthesized amphiphilic copolymers was calculated from ^1^H NMR spectra (Figure 3a). The comparison of sum integrals of aromatic protons shifts at 6.5–7.5 ppm (5*H*, marked as c) and oxymethylene groups shift of PEO_5_MEMA at 4.1 ppm (2*H*, marked as f) allows precise calculation of the copolymer composition by the following equation:

(1) $$F_{1}=\frac{5\times \int \left(4.1\right)}{5\times \int \left(4.1\right)+2\times \int \left(6.5-7.5\right)}$$

where *F*_1_ is the molar fraction of the units of PEO_5_MEMA in a copolymer; chemical shifts at 4.1 and 6.5–7.5 ppm in ^1^H NMR spectra are assigned to oxymethylene group signals of the PEO_5_MEMA (2*H*) and aromatic protons (5*H*) of styrene, respectively; and ∫ are integrals of these signals.

The RAMAN and FTIR spectra of *p*(St-*co*-PEO_5_MEMA) with some different compositions are presented in Figure 3b,c, respectively. The results obtained from RAMAN and FTIR spectra perfectly complement the ^1^H NMR results. The increased intensity of typical aromatic ring or aromatic ring stretch maintain shifts in RAMAN spectra at ca. 1590 and 980 cm^−1^, respectively, is observed as the amount of styrene in the initial polymerization mixture is increased. Meanwhile, the reduction of PEO_5_MEMA in the initial monomer feed directly affects the intensity of typical >C=O absorption bands at ca. 1730 cm^−1^ in FTIR spectra.

**Figure 4:**
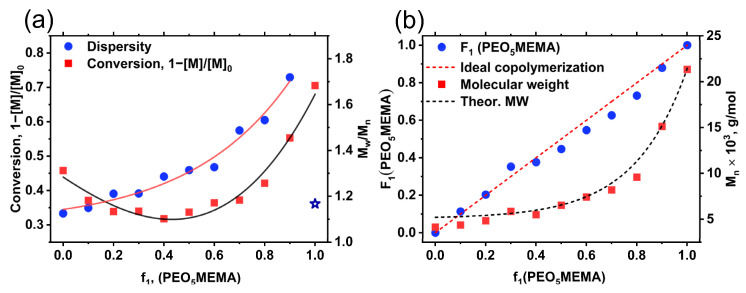
Monomer conversion and dispersity (*M_w_/M_n_*) (**a**); number molecular weight (*M_n_*) and PEO_5_MEMA amount in final composition (*F*_1_) of *p*(St-*co*-PEO_5_MEMA) copolymers (**b**) as a function of PEO_5_MEMA amount in initial molar feed (f_1_) of polymerization mixture. Black and red dashed lines in Figure 5b represent the theoretical molecular weights of *p*(St-*co*-PEO_5_MEMA) copolymers and the monomer distribution in case of ideal copolymerization, respectively.

The dependences of the monomer conversion (determined gravimetrically) and the dispersity of synthesized *p*(St-*co*-PEO_5_MEMA) copolymers, as a function of the PEO_5_MEMA amount in the initial molar feed (f_1_), are presented in Figure 4a. Interestingly, both the monomer conversion and dispersity of the obtained polymeric products are dependent on the initial monomer feed. The lowest yields of copolymers are obtained using an equimolar monomer ratio in the initial polymerization feed. Meanwhile, the dispersity of synthesized polymers increases with increasing the amount of PEO_5_MEMA in the initial molar feed (Figure 4a). Interestingly, this behavior is not related to PEO_5_MEMA polymerization control during the RAFT process because the RAFT homo-polymerization of PEO_5_MEMA is well controlled and results in products of low dispersity (*Ð* = 1.17) (marked as a star in Figure 4a).

**Figure 5:**
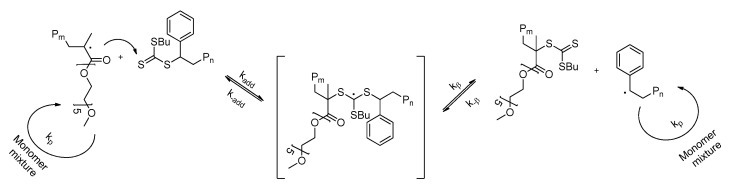
The main equilibrium of RAFT copolymerization of styrene and PEO_5_MEMA.

The remaining question is why the conversion of monomers and the dispersity of synthesized *p*(St-*co*-PEO_5_MEMA) copolymers is affected by the composition of monomers in the feed if it is known that both styrene and PEO_5_MEMA belong to the class of more activated monomers [33]. We suppose that the answer to this question lies in the main equilibrium of the RAFT process (Figure 5). It is known that the reaction of growing macro-radicals with a monomer determines the rate of polymerization [32]. In contrast, the reaction of the macro-radicals with dormant chains, containing terminal trithiocarbonate groups (macro-CTA), provides the “livingness” or controlled character of the system [34]. The latter interaction, during which the intermediate radical compound is formed, is called the main equilibrium of the RAFT process. In the case of copolymerization of styrene and PEO_5_MEMA, all polymerization parameters are related to such intermediate compound formation and fragmentation preferences. The fragmentation of intermediate compound to the secondary radicals having terminal styrene units is slower if compared to tertiary radicals formed from PEO_5_MEMA. Due to PEO_5_MEMA, better radical leaving group ability of the methacryloyl radical leads to it having a higher concentration during a RAFT copolymerization and a higher possibility of chain extension from the macro-radical with PEO_5_MEMA unit on end. If we use the equimolar ratio of monomers in the initial feed, the possibility of different macro-radical (secondary and tertiary) formations that are attached to both sides of the trithiocarbonate compound (formed dormant chain) (Figure 5) is the highest. The higher ratio of PEO_5_MEMA to styrene in the initial polymerization feed is used, the more distorted equilibrium is obtained. In this case, the propagation of chains is preferable from PEO_5_MEMA radical’s side, leading to poorer control of RAFT process and high dispersity of products (Figure 4a).

Figure 4b has demonstrated the dependence of determined copolymer composition (*F*_1_) (calculated from ^1^H NMR spectra) as a function of initial monomer feed (f_1_) in the polymerization mixture. Despite the different monomer propagation rates (as described above, the k_p_^app^ of PEO_5_MEMA in RAFT polymerization rate is more than two orders of magnitude higher if compared to styrene) [34], the compositions of synthesized copolymers were similar to the compositions of monomers in the initial feed. Moreover, the determined molecular weights of *p*(St-*co*-PEO_5_MEMA) copolymers have shown a good correlation with the theoretically calculated ones (see Table S1 in ESI). Please note that the conversion (practical yield presented in Figure 4a) of products was taken into account during these theoretical calculations. These results lead to a question: why these monomers behave as in almost ideal copolymerization if they are so different? We assume that styrene and PEO_5_MEMA RAFT copolymerization is azeotropic. It means that the reactivity ratios of both monomers (r_1_ and r_2_) are lower than unity. There are numerous papers describing synthesis and investigation of block polymers consisting of styrene and PEG methacrylates [37,38] published in the literature; however, there are no publications describing RAFT copolymerization of such monomers. Despite this fact, the free radical polymerization of monomers with a similar structure was extensively studied earlier. Lewis et al. [39] carried out a pioneer work in determining reactivity ratios of various pairs of monomers, including a pair of styrene and methyl methacrylate (MMA). The calculated values for styrene and MMA were 0.5 ± 0.025 and 0.46 ± 0.026, respectively. Mayer [40] in his detailed studies explained that the low reactivity ratio of styrene and MMA is related to preferability for the cross-polymerization rather than homo-polymerization [40]. The practically determined and calculated reactivity ratios of styrene (r_1_) and PEO_5_MEMA (r_2_) using non-linear least square and Kelen–Tüdös linearization methods are presented in Table 1. In the case of styrene/PEO_5_MEMA, the determined reactivity ratio of styrene (r_1_) is higher if compared to the reactivity ratio of this monomer determined in styrene/MMA polymerization [40]. This is explained by the fact that PEO_5_MEMA is even more preferable for cross-polymerization than MMA. This assumption may be justified by recent studies of Boulding et al. [41], where authors stated that the reactivity ratio of MMA is higher if compared to the used PEG-methacrylate during the free radical polymerization of such monomers.

### 3.3. Thermoresponsivity of Amphiphilic Copolymers

Various thermoresponsive polymers have recently been increasingly investigated for possible application in nanotechnology and biotechnology [16,42]. Among them, water-soluble polymers are especially interesting, since they exhibit a lower critical solution temperature (LCST) in water—a potentially useful feature for many biomedical applications. So far, poly(*N*-isopropylacrylamide) (pNIPAM), which displays an LCST in the water around 32 °C, has been the most studied thermoresponsive polymer [17]. This paper demonstrates that entirely different composition *p*(St-*co*-PEO_5_MEMA) copolymers can also give LCST close to physiological temperature, which can be freely changed and adapted by composition change. Moreover, non-linear PEG-containing polymeric compounds have more advantages against pNIPAM. First, the PEGs are known as bio-inert materials that could hide from the immune system and could avoid opsonization in blood [43]. Furthermore, the PEGylation process is the most widely used for modification of various hydrophobic derivatives in order to improve their stability in aqueous solutions and prolong their circulation in the bloodstream [44]. Since 1977, when PEG was attached to bovine serum albumin and liver catalase proteins, various studies demonstrated that PEG efficiently improves drug delivery [45]. Most of the hydrophobic drugs have aromatic substituents in composition. For instance, chlorin-e6, a well-known photosensitizer producing singlet oxygen and widely used in cancer treatment, has porphyrin structure [46]. Therefore, the incorporation of hydrophobic substituents (i.e., styrene) in copolymers composition could improve the immobilization of hydrophobic drugs via *π–π* interactions.

**Figure 6:**
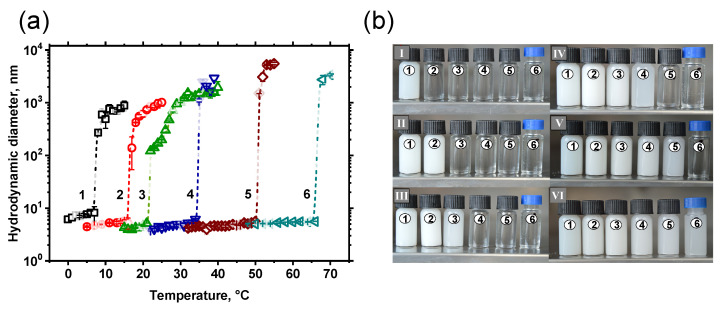
Changes in the hydrodynamic parameters under applied temperature of *p*(St-*co*-PEO_5_MEMA) copolymer with different styrene content (mol.%) in composition (**a**): 55.4 (1), 45.3 (2), 37.4 (3), 26.9 (4), 12.1 (5) 0 (6). The visual turbidity of the samples at different temperatures (**b**): 7 °C (I), 17 °C (II), 26 °C (III), 32 °C (IV), 49 °C (V), 69 °C (VI).

It is also known that pPEO_5_MEMA belongs to thermoresponsive polymers (LCST 61 °C) [28]. Besides, it is extremely soluble in aqueous solutions, whereas styrene is insoluble in aqueous solutions at any temperature. The thermoresponsive properties of *p*(St-*co*-PEO_5_MEMA) with various styrene concentrations were evaluated by measuring the changes of hydrodynamic diameter (*D_h_*) of copolymers in diluted (10 mg/mL) aqueous solutions under exposure to a different temperature. In terms of *p*(St-*co*-PEO_5_MEMA) copolymers, samples with high styrene monomeric units in composition (>63 mol.%) were also insoluble in aqueous solutions. In comparison, the sample with a relatively high styrene content (62.4 mol.%) enabled the formation of a clear solution at 0 °C. Unfortunately, we were not able to determine the changes in hydrodynamic diameter (*D_h_*) values due to technical limitations of the dynamic light scattering apparatus. For this reason, the changes in *D_h_* and LCST of *p*(St-*co*-PEO_5_MEMA) copolymers were measured for copolymers with lower styrene content (0–55.4 mol.%). The *D_h_* and LCSTs were determined by performing DLS measurements every 1 °C within the phase transition temperature range with a temperature stabilization time of 5 min. The DLS measurements were performed cyclically, and no changes in LCST were detected. It confirms that the conformational changes of *p*(St-*co*-PEO_5_MEMA) copolymers were completely reversible. Figure 6a shows the dependence of *D_h_* values as a function of both the temperature and composition of *p*(St-*co*-PEO_5_MEMA) copolymers.

Before the temperature-induced self-assembly, the hydrodynamic radius of macromolecules in all cases is less than 10 nm. However, at LCST copolymers form aggregated structures with a hydrodynamic diameter of few microns; *D_h_* values revolve around 10^3^ nm for styrene rich copolymers and increase close to 10^4^ nm with increasing PEO_5_MEMA content. The phase transition of transparent polymer solutions to opaque under heating can be explained by the disruption of H-bonds between PEO_5_MEMA side chains groups of the polymer and water leading to the formation of micellar aggregates. A sharp decrease in transmittance can be observed in Figure 6b around the LCST, proving the starting point of the copolymers’ precipitation. It is evident that the balance between hydrophilicity/hydrophobicity of the copolymers is shifted toward hydrophilicity with increasing PEO_5_MEMA quantity because of the increasing number of hydrogen-bonding interactions between the water molecules and the copolymers.

**Figure 7:**
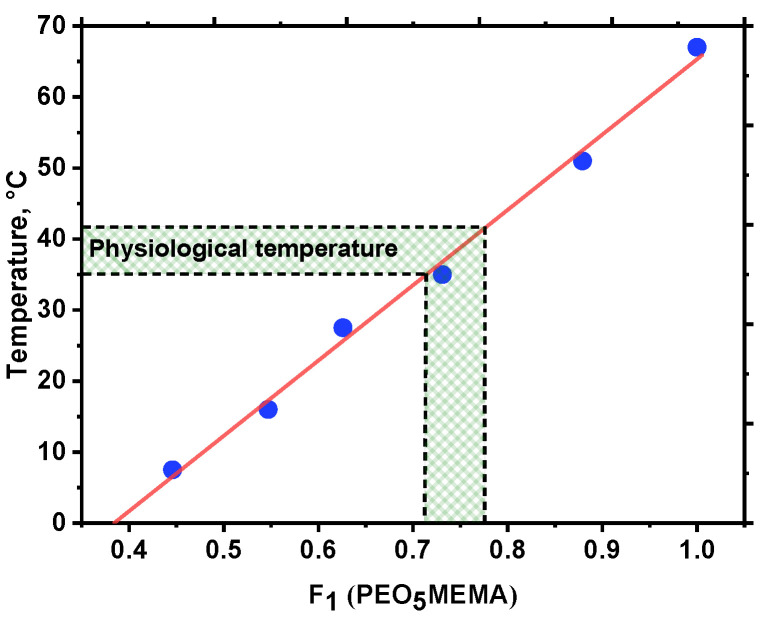
Lower critical solution temperature as a function of *p*(St-*co*-PEO_5_MEMA) copolymers composition.

Another important feature of *p*(St-*co*-PEO_5_MEMA) copolymers is that such clear/cloudy solution transformation is completely reversible, and the LCST is independent of the amount of applied heating/cooling cycles [29,47]. Moreover, it is important to emphasize that the LCST temperature of *p*(St-*co*-PEO_5_MEMA) copolymers is linearly dependent on hydrophilic/hydrophobic monomer ratio in copolymers composition and could be easily adjusted by simply changing the initial monomer feed in the polymerization mixture (Figure 7). The LCST of copolymers with a relatively low content of styrene (ca. 25 mol.%) is in the physiological temperature range; thus, such copolymers could be potentially used as drug delivery systems in bio-fields.

## 4. Conclusions

Well-defined homo styrene and statistical copolymers carrying PEO and styrene chains were synthesized by RAFT copolymerization in the presence of the second generation RAFT CTA 4-(((butylthio)carbonothioyl)thio)-4-cyanopentanoic acid. RAFT copolymerization was well controlled, yielding copolymers with relatively low dispersity (*Ð* 1.15–1.70), desired composition, and a broad range of molecular weights (*M_n_* 2000–21,000 g/mol) with a good correlation with theoretically calculated values.

In this study, we showed that the styrene conversion is dependent on its concentration in the initial feed. Furthermore, the polystyrene synthesis from more concentrated mixtures yielded polymers with higher dispersity. During the extensive studies of styrene and PEO methacrylates copolymerization, we have observed that the composition of copolymers *p*(St-*co*-PEO_5_MEMA) was very close to the composition of corresponding initial monomer feeds, regardless of the achieved conversion. This indicates that both monomers behaved as in the case of ideal copolymerization even though their activity is different. In addition to that, their polymerization is azeotropic. This was confirmed by the calculated reactivity ratios of both styrene and PEO_5_MEMA by non-linear least squares, Kelen–Tüdös, and Kelen–Tüdös at high monomers conversion linearization methods. The obtained results showed that *p*(St-*co*-PEO_5_MEMA) copolymers, synthesized by RAFT copolymerization in 1,4-dioxane, were mostly statistical without any gradient.

Most importantly, the LCST temperature of *p*(St-*co*-PEO_5_MEMA) copolymers is linearly dependent on hydrophilic/hydrophobic monomer ratio in copolymers composition and could be easily adjusted by simply changing the initial monomer feed in the polymerization mixture. The LCST of copolymers with a relatively low content of styrene (ca. 25 mol.%) is in the physiological temperature range; thus, such copolymers could be potentially used as drug delivery systems in bio-fields.

## Figures and Tables

**Table 1:** Reactivity ratios for the copolymerization of styrene/PEO_5_MEMA using the non-linear least squares method, Kelen–Tüdös and Kelen–Tüdös at high monomers conversion linearization methods.

Method	r_1_ (Styrene)	r_2_ (PEO_5_MEMA)	r_1_ × r_2_
Non-linear least squares	0.83 ± 0.06	0.66 ± 0.06	0.55
Kelen–Tüdös	0.81 ± 0.03	0.62 ± 0.05	0.50
Kelen–Tüdös (high q)	0.76 ± 0.03	0.51 ± 0.02	0.39

## Data Availability

The data presented in this study are available on request from the corresponding author.

## Supplementary Materials

The following are available online at www.mdpi.com/article/10.3390/polym14020229/s1. Figure S1-1: ^1^H NMR spectrum of 4-(((butylthio)carbonothioyl)thio)-4-cyanopentanoic acid (BCPA) in CDCl_3_ at 22 °C, Figure S1-2: ^13^C NMR spectrum of 4-(((butylthio)carbonothioyl)thio)-4-cyanopentanoic acid in CDCl_3_ at 22 °C. Figure S2: Simplified mechanism of styrene RAFT polymerization. Figure S3: The MWD curves obtained from SEC analyses for polystyrene synthesized using different monomer concentration in initial feed (T = 80 °C, t = 24 h, [M]0:[CTA]0:[I]0 = 300:3:1). Figure S4: MWD curves of polystyrene synthesized at different [M]0 to [I]0 ratio in initial feed (T = 80 °C, t = 24 h, [CTA]0:[I]0 = 3, [M]0 = 20%). Figure S5: MWD of polystyrene samples quenched after certain time of RAFT polymerization process (T = 80 °C, [M]0:[CTA]0:[I]0 = 300:3:1, [M]0 = 20%). Table S1: Macromolecular results of synthesized *p*(St) (1); *p*(PEO_5_MEMA) (11) and *p*(St-*co*-PEO_5_MEMA) copolymers with various compositions (2–10).
