# Supplementary material for: Synthesis and Study of Thermoresponsive Amphiphilic Copolymers via RAFT Polymerization
Source: Polymers (Basel). 2022 Jan 6;14(2):229. doi: 10.3390/polym14020229 (PMC8777995; doi:10.3390/polym14020229)
Supplement: Supplementary file 1 [file polymers-14-00229-s001.zip › polymers-1520082-supplementary.pdf]

polymers

Supporting information

Synthesis and Study of Thermoresponsive Amphiphilic Copolymers via RAFT Polymerization

Marija Kavaliauskaite, Medeina Steponaviciute, Justina Kievisaite, Arturas Katelnikovas and Vaidas Klimkevicius *

Institute of Chemistry, Vilnius University, LT-03225 Vilnius, Lithuania; ma.kavaliauskaite@gmail.com (M.K.); medeina.steponaviciute@chf.vu.lt (M.S.); justinakevisaite@gmail.com (J.K.); arturas.katelnikovas@chf.vu.lt (A.K.)

* Correspondence: vaidas.klimkevicius@chf.vu.lt; Tel.: +37-066242603

S1. Synthesis of 4-(((butylthio)carbonothioyl)thio)-4-cyanopentanoic acid (RAFT CTA)

4-(((Butylthio)carbonothioyl)thio)-4-cyanopentanoic acid (BCPA) was synthesized according to the general procedure described previously [1] with few adjustments. To a cold (0 °C) solution of 1-butanthiol (2.89 g, 32.0 mmol) in anhydrous diethyl ether (70 mL) 1.45 g NaH (60% in mineral oil, 36.3 mmol) was continuously added. After 30 min of stirring, 12.18 g of CS_2 (160 mmol) was added dropwise at 0 °C. The cloudy yellow reaction mixture, containing the formed sodium butyl carbonotrithionate, was allowed to warm to room temperature. Later on, the mixture was purged with N_2 gas for 30 min, then 5.08 g of iodine (20 mmol) was added in one portion and the reaction mixture was stirred for 1 h at room temperature. The formed precipitate of insoluble NaI in diethyl ether was removed by filtration and the filtrate was washed several times using 1 M $Na_2S_2O_3$ aqueous solution to remove unreacted iodine. The combined organic layers were dried with $MgSO_4$ and the solvent was removed under reduced pressure giving 6.0 g of yellow viscous oil of bis(butyltrithiocarbonate). Subsequently, 4,4-azobis(4-cyanovaleric acid) (ACVA) (13.44 g, 48.0 mmol) was added into three-neck round-bottom flask containing 6.0 g of bis(butyltrithiocarbonate) dissolved in 150 mL of EtOAc, and the solution was stirred overnight under reflux in N_2 atmosphere. The solution was washed with water (3 × 100 mL) to remove unreacted ACVA, and concentrated using rotary evaporator. The product was purified using flash column chromatography (eluent hexane:EtOAc:AcOH = 4:1:0.01 (*v*/*v*), R_f 0.2), and the solvent was removed resulting in yellow solid 4-(((butylthio)carbonothioyl)thio)-4-cyanopentanoic acid. Overall yield 8.29 g (89%).

Analytical data of BCPA: m.p. 41-45 °C; ^{1}H NMR (400 MHz, $CDCl_3$) ppm: 0.96 (t, *J* = 7.4 Hz, 3H), 1.45 (m, 2H); 1.71 (m, 2H), 1.91 (s, 3H), 2.36–2.60 (m, 2H), 2.71 (t, *J* = 7.8 Hz 2H), 3.36 (t, *J* = 7.5 Hz, 2H); ^{13}C NMR (100 MHz, $CDCl_3$) ppm: 13.59, 22.10, 24.86, 29.49, 29.70, 33.48, 36.78, 46.20, 118.90, 177.12, 216.79.

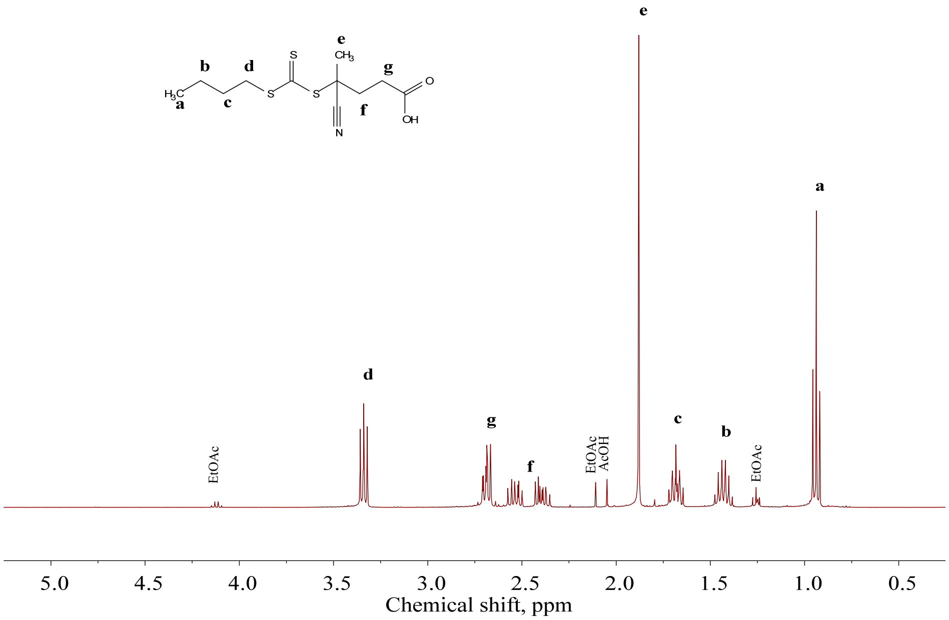

Figure S1–1. ^{1}H NMR spectrum of 4-(((butylthio)carbonothioyl)thio)-4-cyanopentanoic acid (BCPA) in $CDCl_3$ at 22 °C.

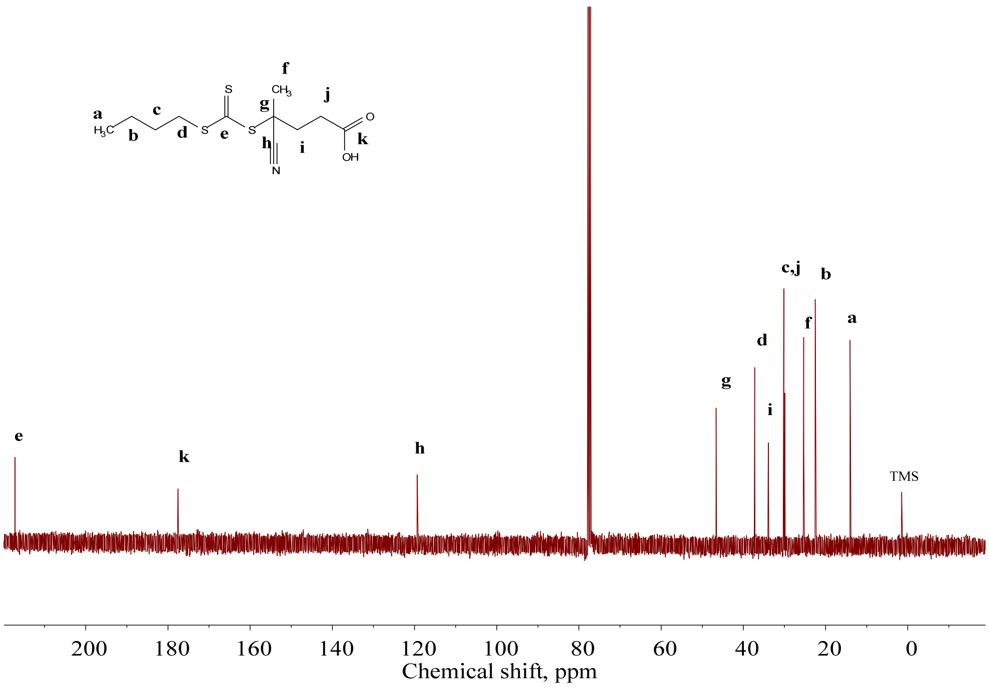

Figure S1–2. ^{13}C NMR spectrum of 4-(((butylthio)carbonothioyl)thio)-4-cyanopentanoic acid in $CDCl_3$ at 22 °C.

S2. Mechanism of styrene RAFT polymerization.

The principal scheme of polystyrene RAFT synthesis is given in Figure S2. The reaction, in our case, was initiated with AIBN as a free radical source. These free radicals ($I^•$) then react with styrene forming short propagating radical chains $P_n^•$ (1) (propagation stage). During the RAFT pre-equilibrium stage, a polymeric radical with n styrene units $P_n^•$ (1) reacts with RAFT CTA forming RAFT adduct radical (2). Subsequently, this RAFT adduct radical (2) can fragment in either direction to yield either starting compounds ($P_n^•$ and CTA) or polymeric RAFT agent P_n–CTA (3) and the additional radical $R^•$ (4) (easily leaving group in CTA. On the re-initiation stage, the newly formed radical $R^•$ (4) reacts with styrene, starting another active polymer chain $P_m^•$ (5). Obviously, the main RAFT equilibrium is the most important stage in RAFT synthesis. During this stage, the newly formed active polymer chain $P_m^•$ (5) could bound either to initial CTA (if still present in polymerization mixture) or to polymeric RAFT agent P_n-CTA (3), forming the new RAFT adduct radical (6), which, in turn, can again fragment in either direction to yield either starting compounds (P_m and P_n–CTA (3)) or new polymeric RAFT agent P_m–CTA (7) and the radical $P_n^•$ (1). When RAFT adduct radical (6) starts to dominate with respect to RAFT adduct radical (2) it is considered that the main RAFT polymerization equilibrium is reached [2, 3] and the propagation probability of polymeric chains is independent on the initial CTA structure anymore. It is also important to mention that different constant values of addition (k_{add}) of macro-radical $P_n^•$ (1) to trithiocarbonate compound (CTA) and fragmentation (k_β) of the formed the RAFT adduct radical (2) highly affect the rate polymerization. Since $k_{add} >> k_\beta$, this causes the retardation of polymerization process, which may last from few minutes to even several hours depending on the chosen CTA [4, 5].

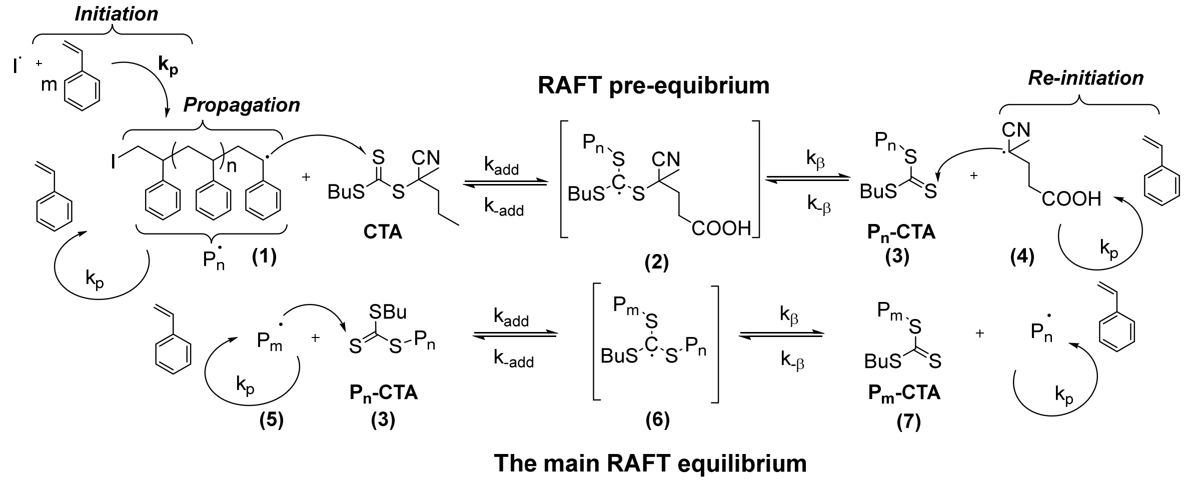

Figure S2. Simplified mechanism of styrene RAFT polymerization.

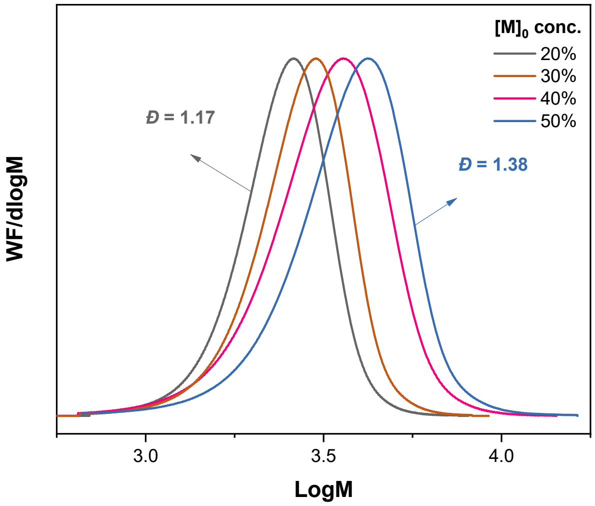

Figure S3. The MWD curves obtained from SEC analyses for polystyrene synthesized using different monomer concentration in initial feed (T = 80 °C, t = 24 h, $[M]_0:[CTA]_0:[I]_0$ = 300:3:1).

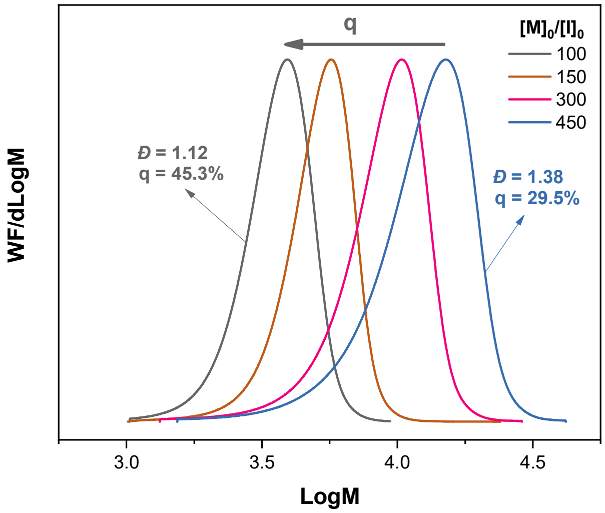

Figure S4. MWD curves of polystyrene synthesized at different $[M]_0$ to $[I]_0$ ratio in initial feed (T = 80 C, t = 24 h, $[CTA]_0:[I]_0$ = 3, $[M]_0$ = 20%).

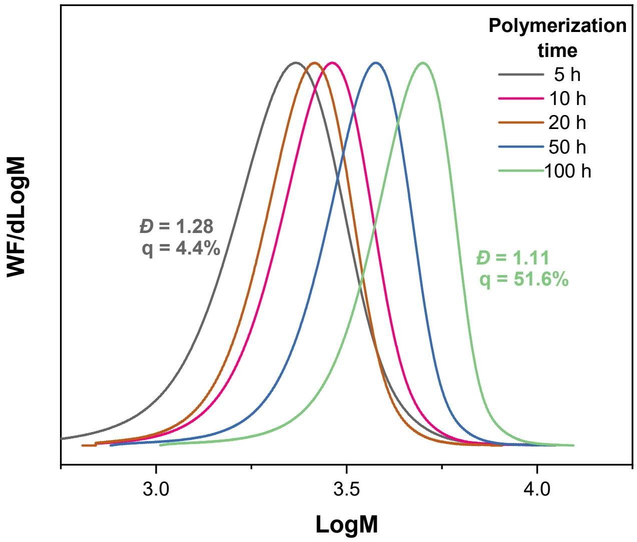

Figure S5. MWD of polystyrene samples quenched after certain time of RAFT polymerization process (T = 80 °C, $[M]_0:[CTA]_0:[I]_0$ = 300:3:1, $[M]_0$ = 20%).

S3. Macromolecular parameters of p(St), p(PEO$_5$MEMA), and p(St-*co*-PEO$_5$MEMA) copolymers

Table S1. Macromolecular results of synthesized p(St) (1); p(PEO$_5$MEMA) (11) and p(St-*co*-PEO$_5$MEMA) copolymers with various compositions (2-10).

No.	[St]:[PEO$_5$MEMA]	$M_{n\ theor}$, g/mol	M_n, g/mol	Đ	Yield q, %
1.	100:0	5060	4130	1.125	45.8
2.	88.7:11.3	4980	4350	1.149	34.1
3.	79.7:20.3	5170	4830	1.211	33.9
4.	64.8:35.2	6180	5850	1.212	34.0
5.	62.4:37.6	5940	5480	1.286	31.8
6.	55.4:44.6	6740	6510	1.314	33.7
7.	45.3:54.7	7980	7410	1.327	36.4
8.	37.4:62.6	8730	8190	1.487	37.2
9.	26.9:73.1	10700	9580	1.532	42.1
10.	12.1:87.9	15570	15110	1.719	55.3
11.	0:100	21410	21340	1.167	70.5

References

1. V. Klimkevicius, M. Steponaviciute, R. Makuska, Kinetics of RAFT polymerization and copolymerization of vinyl monomers by size exclusion chromatography, *Eur. Polym. J.* **2020**, *122*, 109356, doi:https://doi.org/10.1016/j.eurpolymj.2019.109356.
2. G. Moad, E. Rizzardo, S.H. Thang, Living radical polymerization by the RAFT process A second update, *Aust. J. Chem.* **2009**, *62*, 1402–1472, doi:10.1071/CH09311.
3. G. Moad, E. Rizzardo, S.H. Thang, Living radical polymerization by the RAFT process a third update, *Aust. J. Chem.* **2012**, *65*, 985–1076, doi:10.1071/ch12295.
4. E. V. Chernikova, S.D. Zaitsev, A.V. Plutalova, K.O. Mineeva, O.S. Zotova, D.V. Vishnevetsky, Control over the relative reactivities of monomers in RAFT copolymerization of styrene and acrylic acid, *RSC Adv.* **2018**, *8*, 14300–14310, doi:10.1039/c8ra00048d.
5. T. Arita, M. Buback, P. Vana, Cumyl dithiobenzoate mediated RAFT polymerization of styrene at high temperatures, *Macromolecules* **2005**, *38*, 7935–7943, doi:10.1021/ma051012d.
